# Effect of Hydrothermal Cooking Combined with High-Pressure Homogenization and Enzymatic Hydrolysis on the Solubility and Stability of Peanut Protein at Low pH

**DOI:** 10.3390/foods11091289

**Published:** 2022-04-29

**Authors:** Jiaxiao Li, Aimin Shi, Hongzhi Liu, Hui Hu, Qiang Wang, Benu Adhikari, Bo Jiao, Marc Pignitter

**Affiliations:** 1 Institute of Food Science and Technology, Chinese Academy of Agricultural Sciences, Key Laboratory of Agro-Products Processing, Ministry of Agriculture and Rural Affairs, P.O. Box 5109, Beijing 100193, China; lijiaxiao721@outlook.com (J.L.); liuhongzhi@caas.cn (H.L.); huhui@caas.cn (H.H.); jiaobo@caas.cn (B.J.); 2 Shaanxi Provincial Land Engineering Construction Group, Xi’an 710075, China; 3 School of Applied Sciences, City Campus, RMIT University, Melbourne, VIC 3001, Australia; benu.adhikari@rmit.edu.au; 4 Department of Physiological Chemistry, Faculty of Chemistry, University of Vienna, Althanstrasse 14, 1090 Vienna, Austria; marc.pignitter@univie.ac.at

**Keywords:** peanut protein, hydrothermal cooking, combined modification, low pH, physicochemical properties, protein structure

## Abstract

A novel method combining high-pressure homogenization with enzymatic hydrolysis and hydrothermal cooking (HTC) was applied in this study to modify the structure of peanut protein, thus improving its physicochemical properties. Results showed that after combined modification, the solubility of peanut protein at a pH range of 2–10 was significantly improved. Moreover, the Turbiscan stability index of modified protein in the acidic solution was significantly decreased, indicating its excellent stability in low pH. From SDS-PAGE (Sodium Dodecyl Sulfate PolyAcrylamide Gel Electrophoresis), the high molecular weight fractions in modified protein were dissociated and the low molecular weight fractions increased. The combined modification decreased the particle size of peanut protein from 74.82 to 21.74 μm and shifted the isoelectric point to a lower pH. The improvement of solubility was also confirmed from the decrease in surface hydrophobicity and changes in secondary structure. This study provides some references on the modification of plant protein as well as addresses the possibility of applying peanut protein to acidic beverages.

## 1. Introduction

Peanut (*Arachis hypogaea* Linn.) is one of the most important oil crops in the world. China’s peanut production accounts for more than 40% of the world’s total production [1], and 50–65% of the total China’s peanut production is used for oil production.

The peanut meal contained more than 50% protein (dry basis) after pressing or removing the oil [2]. It is estimated that at least 3.5 million tons of defatted peanut meal is utilized in China each year, which is equivalent to approximately 2 million tons of peanut protein. Peanut protein is currently the third largest source of plant protein in China after wheat and soybean [3,4]. Serious protein denaturation happens after the traditional oil extraction process, which results in a huge waste of protein resources. Wang Qiang’s group identified that high-quality peanut protein with a low degree of modification (the nitrogen solubility index is above 70%) could be obtained via improving defatting process [1,5]. Hence, peanut protein extracted from the wasted oil meal by this process is a high-quality and cheap protein source for food industries.

In the recent decade, peanut protein has received increasing attention because of its large output and high nutritional value [6]. At the present, peanut protein with high commercial value can be used as powered ingredient for direct use in high-protein foods and as food additives in foods such as pasta, meat products, and milk powder to improve appearance, water retention, texture characteristics and flavor, and nutritional value of foods [7,8]. Compared with soy protein, the utilization of peanut protein is still relatively low, primarily due to the relatively high cost of production, low solubility, and other characteristics [3]. As stated above, better use of peanut meal can take full advantage of valuable resource and increase the profits of the vegetable protein industry. Therefore, it is an important topic for peanut processing enterprises to further enhance the utilization of peanut protein.

Acidic beverages account for 60–70% of soft drink products and are popular with consumers because of their good taste [9]. However, due to their simple composition and lack of protein, they cannot meet the needs of consumers today for nutrition balance [10,11,12]. The addition of vegetable protein to traditional acidic beverages not only enhances the flavor of the product, but also increases the protein content. Thus, increasing protein content in acidic beverages and making them more stable in acidic beverages are research topics of high interest [13].

The isoelectric points of plant proteins from peanut, soy, pea, and walnut proteins fall under acidic pH range, which is also the pH range of most acidic beverages (pH 3.0 to 4.5). Plant proteins either flocculate or precipitate at their isoelectric point, limiting their use in acidic beverages [14]. The existing vegetable protein-based beverages on the market are mostly neutral or alkaline and have low protein content. Thus, the question of how to expand the application of vegetable protein in acidic beverages is currently a challenge and is an important issue regarding application of plant proteins. At present, methods such as adding polysaccharide stabilizers such as pectin, seaweed, and xanthan gums are used in beverages [15]. These additives increase the viscosity of the beverages and stabilize them by inhibiting the precipitation of protein particles. However, when beverages stabilized in this way are stored for a long time, they are susceptible to phase separation and/or precipitation, which affect their quality [16]. Therefore, the modification of natural plant proteins to achieve their stability in acidic conditions is of great practical significance. It is important to develop a green and efficient plant protein modification method.

Due to the limitations of a single physical, chemical, and enzymatic modification method, this study used a combination of high-pressure homogenization, enzymatic hydrolysis, and hydrothermal cooking to modify peanut protein. High pressure homogenization is commonly used in the food industry. The high-pressure homogenization process simultaneously involves the processes of cavitations, shear, turbulence, and temperature rise [17]. Before the enzymatic hydrolysis of the protein, the high-pressure homogenization pretreatment can loosen the protein structure, break the disulfide bond of the protein, expose more enzyme cleavage sites, and enable the protease to better act on the peptide bond, thereby breaking peptide bonds and accelerating protein breakdown [18].

Hydrothermal cooking is a steam injection process, commonly known as jet cooking, which combines high temperature and shearing force. The HTC equipment is mainly composed of a water heater, a material maintenance pipe, and a cooling system. High-speed turbulent materials mix with compressed air and high-temperature steam passing through the nozzle. Due to the sudden release of pressure in the flash chamber, there is a fast transfer of heat energy, and the temperature of the material rises sharply; the material enters the cooling system after the temperature is maintained for a certain period. This processing method has been used to improve protein extraction and re-functionalize heat-denatured soybean protein in extruded meals [19,20]. HTC can increase the solubility of protein by improving the swelling of protein particles and dissolving the protein aggregates of protein particles, so that the protein has better functional properties. The study of the Xia et al. (2012) [21] proved that HTC improved the extraction rate and purity of the protein in rice bran, as well as improved the solubility of rice protein, providing theoretical support for the industrial application of plant-insoluble protein extraction by HTC technology. The functional properties of protein have been improved after HTC treatment, which may be attributed to the expansion and exposure of protein structure and the increased of protein hydrophilicity.

In this study, the physicochemical properties of peanut protein through the treatments of HPH, HPH-E, and HPH-E-HTC were characterized, including solubility, zeta potential, secondary structure and average droplet size, and the stability and aggregation status. The systematic analysis of the properties of peanut protein can provide a scientific basis for its comprehensive development and utilization in the future.

## 2. Materials and Methods

### 2.1. Materials

Low-temperature defatted peanut protein powder was obtained from Qingdao Changshou Food Co. Ltd. (Qingdao, China). We purchased Neutrase, standard protein marker, and bovine serum albumin (BSA, Standard Grade) from Solarbio Company. (Beijing, China). Neutrase was used in its original form without further purification, and according to the manufacturer its activity was 1000 U/g. The 1, 8-anilinonaphthalenesulfonate (ANS) was obtained from Cayman Chemical Company (Ann Arbor, MI, USA).

### 2.2. Preparation of Modified Peanut Protein

The combined modification of peanut protein was performed in an order of high-pressure homogenization, enzymatic hydrolysis, and hydrothermal cooking. First, 7% (*w*/*v*) peanut protein aqueous solution was homogenized using a nano homogenize machine (AH-100D, ATS Engineering Limited Company, Shanghai, China) under the pressure of 800 bar for 20 min. Half of this homogenized sample (HPH) was freeze-dried and stored at 18 °C before analysis. The remaining HPH protein sample was treated by enzymatic modification at pH 7.0. According to the manufacturer instructions, Neutrase was added to start the hydrolysis at 50 °C for 50 min. Once the hydrolysis was complete, the hydrolysates were heated to 85 °C for 10 min to inactivate the enzyme. Half of this HPH-E sample was freeze-dried and stored at 18 °C before analysis. The final HPH-E-HTC sample was prepared by subjecting the remaining HPH-E sample into a hydrothermal cooking treatment using the HTC system (ESCS-M104, Xiaoledongchao Food Machine Company (Co. Ltd., Shanghai, China) at 130 °C for 120 s and then cooled to 25 °C. The obtained HPH-E-HTC sample was freeze-dried and preserved at 4 °C before analysis. The natural peanut protein was used as comparison.

### 2.3. Determination of Protein Solubility

A 1% (*w*/*v*) protein solution was prepared with distilled water and the pH was adjusted to 2.0–10.0 with 0.5 M HCl or 0.5 M NaOH. After stirring for 40 min, each sample was centrifuged at 10,000× *g* for 10 min at 20 °C. The protein content of supernatant was measured by Lowry’s method [22] (Lowry, Rosebrough, Farr, and Randall 1951) and estimated from the abovine serum albumin (BSA) calibration curve (r^2^ = 0.9994). The results were expressed as grams of soluble protein/100 g of protein used.

### 2.4. Determination of Main Protein Fractions

SDS-PAGE experiments were performed with a precast 5~13% gradient polyacrylamide gel according to the previously reported method by Guo et al. [23]. Briefly, 4 mg protein sample was dissolved in 1.0 mL of loading buffer and then heated in boiling water for 10 min. After centrifugation (4500× *g*, 10 min), 8 μL of supernatant was loaded into the gel. Electrophoresis was performed under 80 V followed by 110 V. Subsequently, the gel was stained with 0.1% (*w*/*v*) Coomassie brilliant blue (R-250) for 2 h and destained with 5% methanol/10% acetic acid/85% deionized water for 12 h. The images were finally taken using a Bio-Rad gel imaging system.

### 2.5. Determining the Stability of Protein Solution

The stability of protein solution was determined by a dispersion stability analyzer (multiple light scattering instrument) (Turbiscan Lab, Formulaction, Toulouse, France). The light source detector scans from the bottom of the sample bottle to the top of the bottle, and the sample scanning range is 2 to 45 mm. The final application software analyzes the graph to obtain the stability index (Turbiscan stability index, TSI). A lower TSI value indicates better stability of the system [24,25].

### 2.6. Measurement of Particle Size

The protein sample was dispersed in distilled water with a solution concentration of 1% (*w*/*v*) and stirred continuously for 2 h until testing. The particle size distribution of protein samples was measured with Mastersizer 3000 (Malvern instruments, Worcestershire, UK). The refractive index of the dispersed phase is 1.570 and that of the continuous phase (water) is 1.333.

### 2.7. Determination of Zeta-Potential

The titer zeta potential of the protein was measured using Nano-ZS and MPT-2 zeta potential and nanometer particle size distribution instrument (Malvern Instrument Ltd., Worcestershire, UK). Protein solution of 0.5% (*w*/*v*) was stirred at 100 rpm under ambient temperature for 1.5 h and the pH was adjusted with 0.25 M NaOH and 0.25 M HCl. Using the test zeta potential function, we set the measurement interval to pH 2.0–10.0. All determinations were made with at least two freshly prepared undiluted samples.

### 2.8. Determination of Surface Hydrophobicity

Surface hydrophobicity (H_0_) was measured using 8-aniline-1-naphthalenesulfonic acid ammonium salt (ANS) as a fluorescent probe. The protein sample to be tested was dissolved in a phosphate buffer solution (0.01 M, pH 7.0) to prepare 1 mg/mL protein dispersion. The supernatant was obtained after centrifugation at 10,000× *g* for 20 min.

The protein content in the supernatant was determined by the Folin phenol protein quantification method. The above protein solutions were then diluted to different concentrations (0.019, 0.038, 0.075, 0.150 mg/mL) using the same buffer. Next, 4 mL of the diluted solution and 20 μL of ANS solution (8.0 mM) were added together and mixed well. The fluorescence intensity was quickly measured with a Hitachi-F-2500 fluorescence spectrophotometer (F-2500, Hitachi Co., Tokyo, Japan). The emission and excitation wavelengths were set to 470 nm and 390 nm, and H_0_ is the slope of the fluorescence intensity versus protein concentration plot.

### 2.9. Determination of Secondary Structure of Protein

FTIR spectroscopic tests were conducted using TRUORS 27 spectrophotometer (TRUORS 27, Buruker, Germany). The spectra were obtained at 60 cm scan at 4 cm^−1^ resolution. The dried samples were tableted onto the ATR accessory of Spectro technology with KBr at 4 cm^−1^ resolution. Each sample was scanned 64 times and each sample was measured 3 times and averaged. The wavelength range in these tests was 500–4000 cm^−1^ scanned at 4 cm^−1^ resolution.

### 2.10. Observing Microstructure

The morphological properties of the protein were measured with a scanning electron microscope (SU8010, Hitachi Science Systems, Tokyo, Japan).

### 2.11. Statistical Analysis

The tests were carried out three times. We used v17.0 SPSS Statistics software (IBM Corp., Armonk, NY, USA) to perform one-way ANOVA on the dataset, and the results were expressed as mean ± standard deviation. The significant difference between the two mean values at 95% confidence level was determined by multiple-range test of Duncan (*p* < 0.05).

## 3. Results and Discussion

### 3.1. Protein Solubility

**Figure 1:**
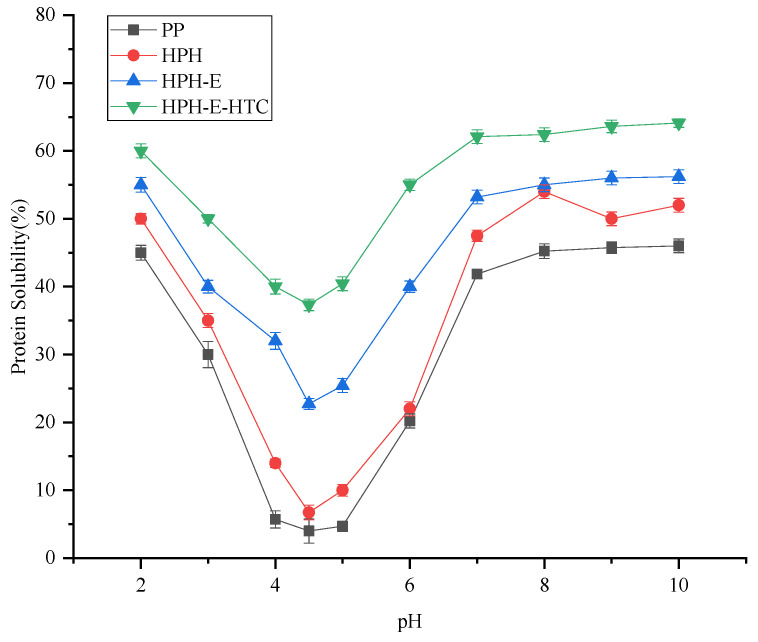
Solubility of peanut protein (PP), high pressure homogenized protein (HPH), high pressure homogenized and enzyme treated protein (HPH-E), and HPH-E-hydrothermally cooked (HPH-E-HTC) protein as a function of pH.

The solubility of untreated peanut protein (PP), high pressure homogenized peanut protein (HPH), HPH and enzyme treated peanut protein (HPH-E), and subjected to all these treatments peanut protein (HPH-E-HTC) in the range pH 2.0–10.0 are presented in Figure 1. The solubility of HPH, HPH-E, and HPH-E-HTC in the range pH 2.0–10.0 were significantly higher (*p* < 0.05) than PP (%). The protein solubility of all samples reached a minimum around pH 4.5; the protein solubility of HPH-E-HTC at pH 4.5 was still 37.3% compared to 4.0% of PP. As pH > 7.0, the solubility of all hydrolysates exceeded 50%, significantly higher than that of PP (*p* < 0.05). The HPH and HPH-E samples exhibited different protein dissolution with changes in pH. The solubility of the PP suspension increased significantly after high-pressure homogenization treatment. The improvement in solubility can be attributed to the fact that turbulence forces and cavitation involved in the high-pressure homogenization had broken down the molecular chain because of order-to disorder transitions, and opened the molecular structure. The mechanical energy supplied by the high-pressure homogenizer breaks down the structure of protein and expose more charged moieties so that the protein can bind better with water, which improves protein solubility [17]. Enzymatic hydrolysis significantly increased the solubility of peanut protein over the entire tested pH range. Solubility of protein can also be improved by enzymatic hydrolysis due to the breaking down of protein to more soluble peptides and increased exposure of ionizable amino and carboxyl groups. The unfolded protein can more readily interact with water through the exposed hydrophilic groups, and the looser tertiary conformation can promote hydration [12]. Therefore, combination of HPH, enzymatic hydrolysis, and HTC can be used as an effective modification technique for improving the solubility of peanut protein.

### 3.2. Morphology of Modified Peanut Proteins

**Figure 2:**
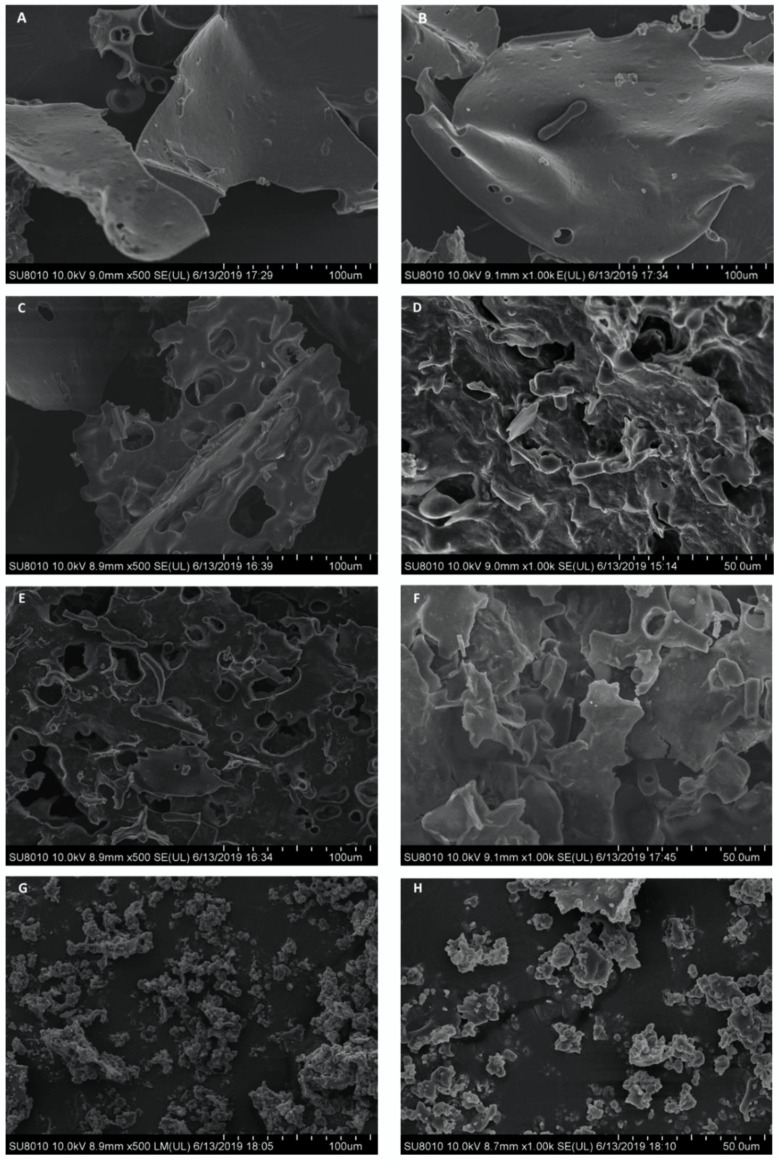
SEM micrographs of peanut proteins obtained through different modification methods: (**A**): peanut protein (PP) ×500; (**B**): peanut protein (PP) ×1 k; (**C**): high pressure homogenized protein (HPH) ×500; (**D**): high pressure homogenized protein (HPH) ×1 k; (**E**): high pressure homogenized and enzyme treated protein (HPH-E) ×500; (**F**): high pressure homogenized and enzyme treated protein (HPH-E) ×1 k; (**G**): HPH-E-hydrothermally cooked (HPH-E-HTC) protein ×500; (**H**): HPH-E-hydrothermally cooked (HPH-E-HTC) protein ×1 k.

Figure 2 shows SEM micrographs of peanut proteins obtained through different modification methods. PP appeared as a sheet-like surface structure with ridge-like protrusions. No large pore-like structure was observed. HPH-PP appeared dense and less porous, whereas the HPH-E-PP became loose and disordered with pores becoming smaller and more ordered after high-pressure homogenization and moderate enzymatic hydrolysis. The surface area of these treated protein appeared large due to very high unevenness. Protein with this structure may disperse in water quite rapidly. The protein morphology was consistent with those of protein after jet cooking shearing and heat treatment in the study of Gong et al. (2016) [3].

### 3.3. SDS–PAGE Analysis

**Figure 3:**
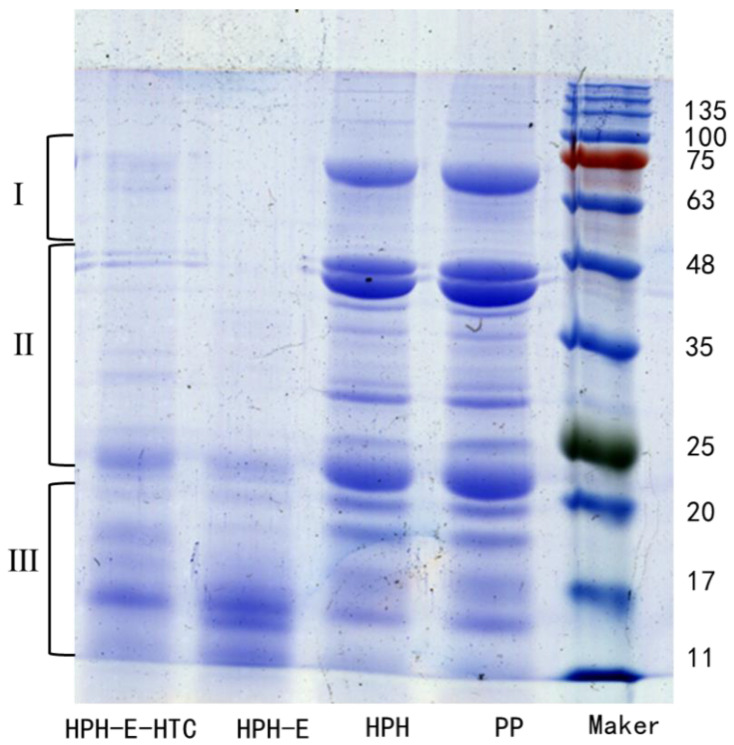
Sodium dodecyl sulphate–polyacrylamide gel electrophoresis profiles of peanut protein (PP), high pressure homogenized protein (HPH), high pressure homogenized and enzyme treated protein (HPH-E), and HPH-E-hydrothermally cooked (HPH-E-HTC) protein.

Figure 3 shows the SDS-PAGE profiles of PP, HPH, HPH-E, and HPH-E-HTC. A typical SDS-PAGE profile of peanut protein consists of 7 s and 11 s fractions [23] and their molecular weight ranges 48.0–245.0 kDa, 20.0–48.0 kDa and 20.0 kDa, representing bands I, II and III, respectively. The SDS-PAGE profile (reducing) observed in this study (Figure 2) is consistent with those previous reported. There are four bands of peanut globulin with molecular weights of 40.5 kDa, 37.5 kDa, 35.5 kDa, and 23.5 kDa. Conarachin I have three bands with molecular weight 18 kDa, 17 kDa, or 15.5 kDa. Similarly, conarachin II has one band with a molecular weight of 61 kDa [26]. All the proteins were electrophoresed with equal amounts of soluble proteins (15 ug), and the result showed that there was no alteration in molecular weight distribution of protein fractions treated with high-pressure homogenization (HPH) showing that HPH treatment did not affect the protein composition. The HPH-E and HPH-E-HTC treated peanut proteins showed significant alteration in all the bands; band I1 with molecular weight 64 kDa and band II with molecular weight 48 kDa completely disappeared, reflecting the complete degradation of these protein fractions. Band II2, having molecular weight about 24 kDa, was significantly altered in HPH-E and HPH-E-HTC treated peanut protein. In contrast, band III3, having molecular weight 15 kDa, 17 kDa, and 19 kDa, increased after HPH-E treatment and then slightly decreased after HPH-E-HTC treatment. SDS-PAGE images also show a light band with molecular weight above 100 kDa in the HPH treated sample, which indicates HPH induced denaturation and aggregation, which is consistent with previous study [27].

Further treatment of HPH treated protein with enzyme did not show aggregated protein bands, and bands II and II also disappeared due to enzymatic digestion. Interestingly, enzyme treatment of HPH treated peanut increased the intensity of band III, suggesting digestion (breakdown) of high molecular weight fractions into smaller ones followed by their aggregation [21]. Further treatment of HPH-E with heat treatment, i.e., HPH-E-HTC, further reduced the intensity of low molecular weight protein fractions slightly.

### 3.4. Physical Stability in Aqueous Solution Analyzed by Turbiscan

**Figure 4:**
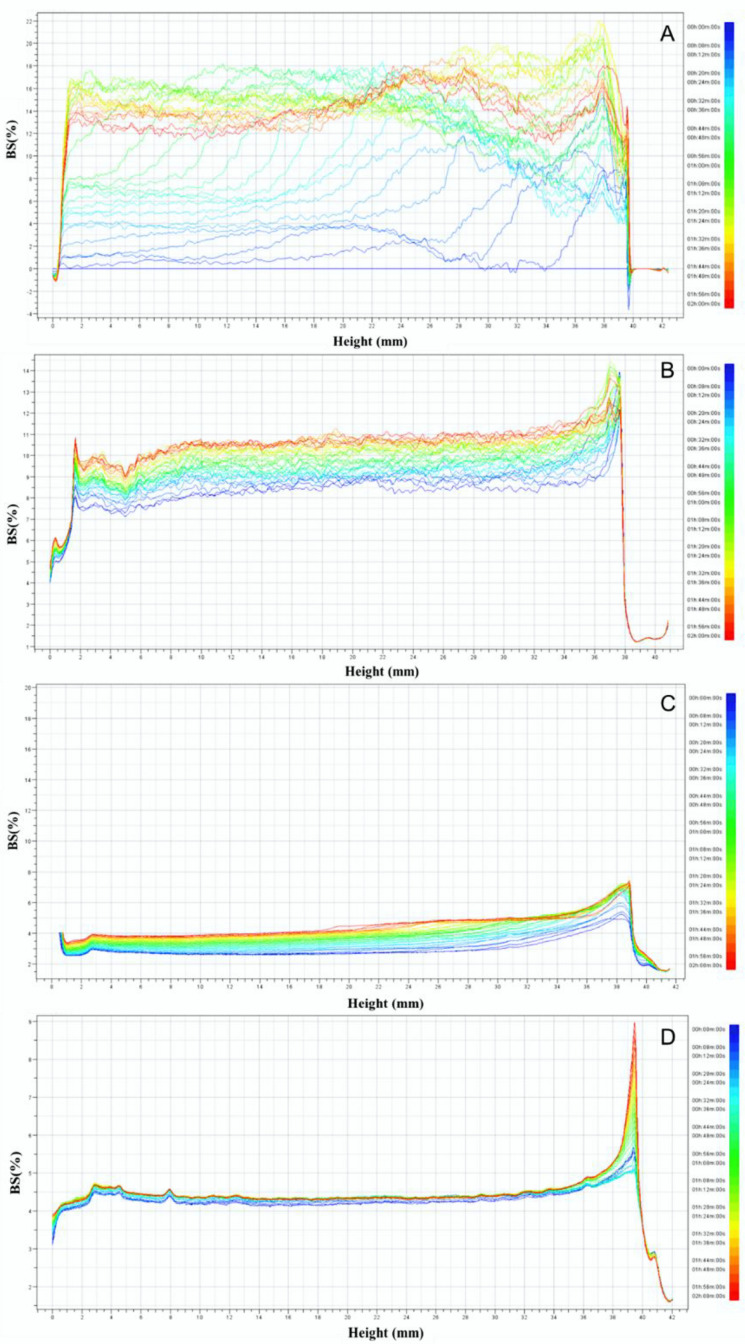
Backscattering (BS) profiles of peanut proteins obtained through different modification methods as a function of the tube height with storage time: (**A**): peanut protein (PP); (**B**): high pressure homogenized protein (HPH); (**C**): high pressure homogenized and enzyme treated protein (HPH-E); (**D**): HPH-E-hydrothermally cooked (HPH-E-HTC) protein.

Figure 4 shows the backscattering (BS) profiles of peanut proteins subjected to HPH, HPH-E, and HPH-E-HTC as a function of tube height with storage time. As can be observed, these treatments had a significant effect on the stability of the peanut protein.

The change of back scattering (ΔBS) with time, as shown in Figure 4A, indicated that there was sedimentation of protein at the bottom indicated by the ΔBS value 4–16% in the 4–34 mm height range. The PP sample had relatively large flocculation and coalescence in the middle and creaming on the top. Figure 4B represents the data for the sample subjected to high pressure homogenization. The ΔBS data fluctuated from 10% to 15% in the 4.00–34.00 mm height range of the HPH treated sample. The ΔBS value of 19% on the top part (36.00–40.00 mm) was the upper peak. The smaller ΔBS value indicates better stability of protein solution, so we can know that the stability of the HPH sample is not good, but the HPH produced more stable solution than the untreated PP. As seen from Figure 4C, at the top portion of the sample (height = 36.00–42.00 mm), ΔBS gradually increased to 7.50%, indicating a slight creaming of protein. There was a convex peak at the top of 36.00–41.00 mm. There was some sedimentation at the bottom and flocculation at top of the HPH-E sample. However, overall, HPH-E was more uniform and more stable than HPH. In Figure 4D, the HPH-E-HTC sample solution was homogeneous, and the fluctuation of ΔBS bottom and middle portion of sample was small, indicating that the precipitation and flocculation of protein particles were not significant. The peak of ΔBS gradually increased to 4.7% in the height range of 0.0 to 0.5 mm. There is a small raised peak at 38~40 mm with ΔBS value of 9.0%, and there was a very small fraction of protein that floated. It is apparent from the figure that the stability of the HPH-E-HTC treated sample was significantly better than that of the untreated sample PP.

**Figure 5:**
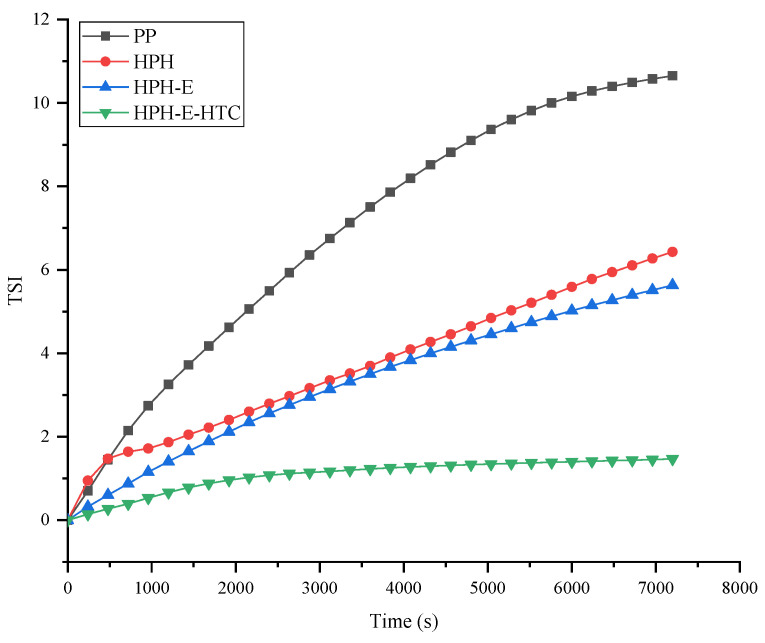
The instability index of peanut protein (PP), high pressure homogenized protein (HPH), high pressure homogenized and enzyme treated protein (HPH-E), and HPH-E-hydrothermally cooked (HPH-E-HTC) protein as a function of time.

Figure 5 shows the Turbiscan stability index (TSI of PP, HPH, HPH-E, and HPH-E-HTC) protein as a function of time. Larger deviation from the starting line (TSI = 0) and higher slope indicate greater instability and vice versa.

The slope of HPH-E-HTC was the smallest and the stability of the solution system was the best, whereas the slope of the PP was the steepest indicating that the stability of its solution was the poorest.

These TSI data shows that the order of stability of these samples was: HPH-E-HTC > HPH > HPH-E > PP, which corroborates their solubility data (Section 3.1) backscattering (BS) profiles. These data indicate that high pressure homogenization plays an important role in stabilizing protein solutions. This is because high pressure homogenization can provide a strong mechanical force that improves the solubility of protein in aqueous medium and make solutions homogeneous. Enzymatic hydrolysis and HTC unfold the protein structure and expose a higher number of hydrophilic groups and thus improves interaction with water. This result is consistent with the surface hydrophobicity data discussed in Section 3.7.

### 3.5. Zeta-Potential

**Figure 6:**
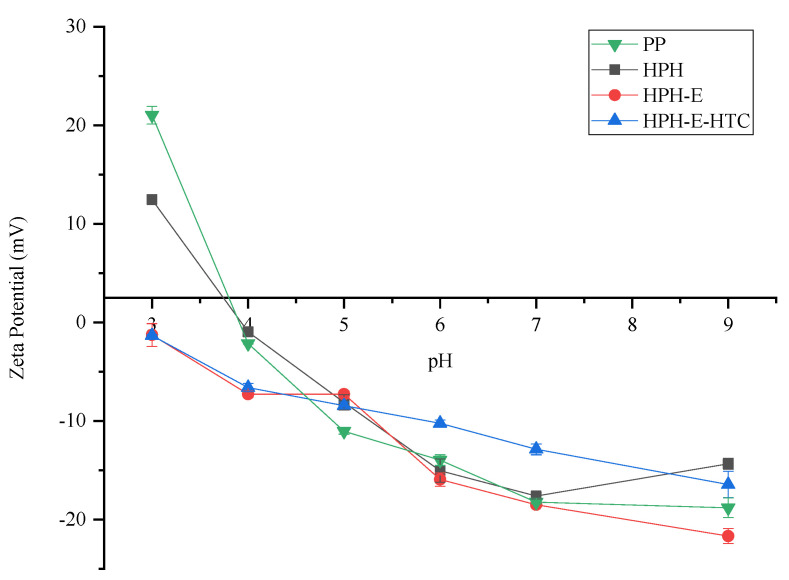
Zeta potential (ζ) profiles of peanut protein (PP), high pressure homogenized protein (HPH), high pressure homogenized and enzyme treated protein (HPH-E), and HPH-E-hydrothermally cooked (HPH-E-HTC) protein as a function of pH at 25 °C.

The relationship between the zeta (ζ) potential of various protein samples as a function of pH is shown in Figure 6. The zeta-potential of PP changed from +21.0 mV to −18.8 mV in the range from pH 2.0 to 10.0. The isoelectric point was observed at pH 3.8. The relationship between zeta-potential and pH are significantly affected by different treatments. There was no significant change in zeta-potential after HPH treatment. The HPH-E and HPH-E-HTC treated proteins were similar with PP at pH > 5, but at pH < 5, the zeta-potential of HPH-E and HPH-E-HTC protein decreased significantly and was negatively charged, and the isoelectric point showed a tendency to migrate to a lower pH. PP has no charge under low pH conditions (near pH = 3.8), so aggregation and precipitation occur between proteins, and water solubility and stability are poor.

The HPH-E and HPH-E-HTC treated proteins are negatively charged under these conditions. The most noteworthy feature of these profiles is that isoelectric point was observed only in native PP and high pressure homogenized PP (HPH), and the HPH-E-HTC and HPH-E were still negatively charged at pH 3.0. This can be attributed to the increased exposure of hydrophilic groups of protein due to high temperature and high pressure [18]. The presence or dominance of charged or polar amino acids on the surface of the aggregate prevents their precipitation, and thus the solubility and stability of protein solubility is improved. This is consistent with previous research on soy protein [25].

### 3.6. Particle Size Distribution

**Figure 7:**
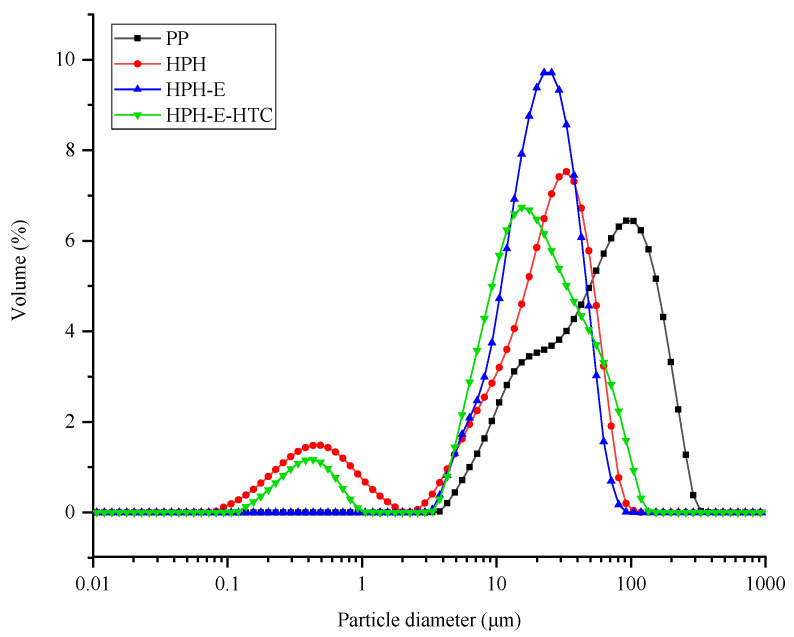
Particle size (D (4,3)) distributions of peanut protein (PP), high pressure homogenized protein (HPH), high pressure homogenized and enzyme treated protein (HPH-E), and HPH-E-hydrothermally cooked (HPH-E-HTC) protein.

Figure 7 shows the particle size distributions of PP, HPH, HPH-E, and HPH-E-HTC, and their volume-weighted mean diameters (D(4,3)) are listed in Table 1.

The D(4,3) value of the untreated peanut protein powder was 74.82 μm. The protein powder prepared by HTC showed significantly (*p* < 0.05) lower D (4,3)value as compared to the control samples.

The extent of decrease in particle size in HTC depended on the temperature and protease pretreatment. Proteolytic pretreatment prior to HTC significantly (*p* < 0.05) reduced the average droplet diameter of the dispersion. The combined modification of peanut protein led to a decrease in the average particle size. Smaller protein particles are easier to disperse uniformly in aqueous solution and less precipitation will happen, resulting in improved solubility.

This may be because the treated peanut proteins show more disordered conformation, leading to increased exposure of hydrophobic domains evidenced by surface hydrophobicity data (Section 3.7). Unfolded proteins are more easily dispersed in water than naturally folded molecules and form a stable dispersion.

When subjected to high-pressure homogenization and moderate enzymatic hydrolysis, the particle size of peanut protein aggregates decreased to a similar extent (24.2 μm and 23.6 μm, respectively). This indicated that high pressure homogenization and moderate enzymatic hydrolysis had similar effects on protein particle size. After HTC treatment, the particle size was reduced to 21.7 μm. This may be caused by high shearing and impact by the HTC process, which destroys the ordered structure and reduces the particle size. After high-pressure homogenization-enzymatic followed by hydrolysis-high temperature cooking, the particles had narrow (more uniform) size distribution.

### 3.7. Surface Hydrophobicity

The change in hydrophobicity (H_0_) of the protein surface is presented in Table 2. The H_0_ of natural untreated peanut protein powder was 644.4. After high-pressure homogenization and combined enzymatic hydrolysis and HTC, it decreased to 297.5. This indicates that high pressure, enzymatic hydrolysis, and HTC can hinder the hydrophobic groups of proteins from being exposed to the surface and reduce surface hydrophobicity, increase hydrophilic groups, and better bind proteins to water.

Similar findings were reported by Xia et al. [21] and Feng et al. [28] in the case of heat-stabilized rice bran prepared using amylase pretreatment combined with hydrothermal cooking amylase pretreatment. The peanut protein is expected to undergo unfolding during these treatments then expose hydrophilic groups and increase interaction with water.

### 3.8. Change in Peanut Proteins’ Secondary Structure

As can be seen from Table 3, the HP-H-E-HTC had the highest β-sheet content (45.9%) among all the modified samples. However, the α-helics and random coil structures showed gradual decrease, and the former decreased the most. The β-turn and β-sheet structures of native PP increased from 14.36% and 25.18% to 25.34% and 45.93% after HP-H-E-HTC treatment, indicating that the α-helical and random coil structures changed to the β-sheet and β-turn structure when subjected to high pressure, high temperature treatment and enzymatic hydrolysis. HPH and HPH-E treated samples also had lower α-helix and random structures, but there was no definite trend in in β-sheet and β-turn structures. The fact that these treatments decreased the α-helical and random coil structures indicated that the protein structure was first unfolded and then reaggregated to improve protein solubility. These results are supported by the research of Blanc et al. [29] and Rao et al. [30], both of which reported a significant increase of β-sheet and decrease of α-helix in the secondary structure of heat-treated peanut protein after boiling or roasting.

## 4. Conclusions

This study shows that the novel combined high-pressure homogenization, enzymatic hydrolysis, high temperature cooking (HPH-E-HTC) treatment significantly improved the solubility and stability of peanut protein solution. Smaller pieces of peanut protein prepared by HPH-E-HTC were observed due to high pressure, enzymatic, and shearing force treatment. The sample prepared by HPH-E-HTC showed lower surface hydrophobicity and particle size and the zeta-potential with negative charge than PP and HPH-E in low pH conditions (near pH = 3.8), indicating improved solubility and stability. These results indicated that HPH-E-HTC can be used to improve the quality functional properties of peanut protein obtained from low-temperature defatted peanut meal. Furthermore, this kind of modified peanut protein has a great potential as a food additive for acidic beverages.

## Figures and Tables

**Table 1:** Particle-size distributions of peanut protein (PP), high pressure homogenized protein (HPH), high pressure homogenized and enzyme (E) treated protein (HPH-E), and HPH-E-hydrothermally cooked (HPH-E-HTC) protein.

Samples	D (4,3) (μm)
PP	74.82 ± 0.57 c
HPH	24.18 ± 0.32 a
HPH-E	23.65 ± 0.39 ab
HPH-E-HTC	21.74 ± 0.17 b

Data are expressed as means ± SD. Superscript characters (a–c) indicate significant (*p* < 0.05) differences in the same column.

**Table 2:** Surface hydrophobicity (H_0_) of peanut protein (PP), high pressure homogenized protein (HPH), high pressure homogenized and enzyme (E) treated protein (HPH-E), and HPH-E-hydrothermally cooked (HPH-E-HTC) protein.

Samples	PP	HPH	HPH-E	HPH-E-HTC
H_0_	644.41 ± 4.68 a	582.67 ± 2.11 b	346.20 ± 1.15 c	297.45 ± 0.75 d

Data are expressed as means ± SD. Superscript characters (a–d) indicate significant (*p* < 0.05) differences in the same row.

**Table 3:** The secondary structure of peanut protein (PP), high pressure homogenized protein (HPH), high pressure homogenized and enzyme (E) treated protein (HPH-E), and HPH-E-hydrothermally cooked (HPH-E-HTC) protein.

Samples	PP	HPH	HPH-E	HPH-E-HTC
Helix	43.41 ± 0.86 a	36.02 ± 1.06 b	21.08 ± 0.85 c	19.72 ± 0.73 d
Beta-turn	14.36 ± 0.78 a	10.92 ± 0.97 b	19.96 ± 0.80 c	25.34 ± 0.53 d
Beta-sheet	25.18 ± 0.46 a	34.82 ± 0.52 b	30.05 ± 0.62 c	45.93 ± 0.47 d
Random	17.05 ± 0.67 a	18.24 ± 0.69 b	28.90 ± 0.76 c	9.00 ± 0.89 d

Data are expressed as means ± SD. Superscript characters (a–d) indicate significant (*p* < 0.05) differences in the same row.

## Data Availability

The datasets generated for this study are available on request to the corresponding author.
